# What kinds of government trust structures affect political participation? Evidence from Chinese Youth Netizens

**DOI:** 10.1371/journal.pone.0323981

**Published:** 2025-05-27

**Authors:** Cheng Wen, Qian Hu, Sheng Chen

**Affiliations:** School of Public Policy and Administration, Chongqing University, Shapingba, Chongqing, China; Rikkyo University: Rikkyo Daigaku, JAPAN

## Abstract

Based on 2018 research data on Chinese netizens’ social awareness, this paper examines how different government trust structures influence youth netizens’ political participation. The findings indicate that the reinforcing effect of government trust on political participation follows a declining order: paradoxical government trust, equal trust, hierarchical government trust, and equal distrust. In this context, subjective well-being is introduced as a mediating variable. The results show that the positive impact of subjective well-being on political participation varies across different trust structures, decreasing in the following order: equal trust holders, paradoxical government trust holders, hierarchical government trust holders, and equal distrust holders. Furthermore, heterogeneity analysis reveals that the negative impact of equal distrust and hierarchical government trust is weaker on online political participation than on offline political participation, while paradoxical government trust has a stronger positive effect online than offline. Overall, the influence of government trust structure is weaker for online political participation than for offline political participation.

## Introduction

[Motivation] In the wave of digitalization, the internet penetration rate is increasing, meanwhile the age of netizens is getting younger and younger. As the main group of internet user, youth netizens realize the interaction between online and offline political participation based on the interactive online platform, because the virtual space expand the ways of youth political participation [1]. Youth is the hope of national development and national rejuvenation, while the youth political participation is full of tension and uncertainty. Therefore, how to make youth political participation active and orderly is an important part of the construction of socialist democracy with Chinese characteristics.

[Research Question] Since the reform and opening up, with economic and social development, Chinese citizens’ participation in political affairs has been expanding and deepening constantly [2]. However, citizens are not born to actively participate in politics, they need external factors [3]. Government trust is one of the key factors promoting citizens’ political participation. Gopalan even declared that without government trust, there is no political participation [4]. At present, China’s citizens generally have a “central-strong-local-weak” trust pattern [5–7]. With the level of Chinese government increasingly rising, the government trust gradually increases [8]. Li calls it as hierarchical government trust [9–12]. On this basis, Lv measures the hierarchical government trust structure by the trust difference between central and local governments and the trust difference between superior and subordinate governments, and classified the government trust structure from poor to excellent into four types [13]. They are paradoxical government trust with “central-weak-local-strong”, equal distrust with “central-weak-local-weak”, hierarchical government trust with “central-strong-local-weak” and equal trust with “central-strong-local-strong”. In the study of government trust structure and political participation, scholars found that the trust gap between central and local governments is an important causal factor leading to citizens’ participation in protests [14]. To further explore the relationship between government trust structure and political participation, Zheng constructs a consistency matrix model of “central-local government trust” and concludes that “central-local government trust” can influence political participation regardless of whether it is consistent or not [15]. By comparing the effects of government trust structure on different political participation, Lv finds that citizens with hierarchical government trust show the most active performance in both electoral political participation and protest political participation [16]. The above studies have laid the foundation for the subsequent exploration of the relationship between government trust structure and political participation. However, scholars have not deeply explored whether the structure of government trust has a differential impact on political participation, nor have they examined the mechanism by which subjective well-being may influence this relationship. These two questions are the focus of this study.

[Methodology] First, a theoretical framework of government trust structure and political participation is constructed based on the theory of rational action, Then, based on the data from the 2018 Chinese Social Awareness Survey of netizens, the OLS model and mediation model are employed for empirical validation.

[Results] The study finds that the reinforcing effect of the paradoxical government trust, equal trust, hierarchical government trust, and equal distrust on political participation gradually weakens. On this basis, subjective well-being is introduced as a mediating variable. The reinforcing effect of the subjective well-being of equal trust holders, paradoxical government trust holders, hierarchical government trust holders, and equal distrust holders decrease from high to low. Finally, heterogeneity analysis reveals that the negative impact of equal distrust and hierarchical government trust is weaker on online political participation than on offline political participation, while paradoxical government trust has a stronger positive effect online than offline. Overall, the influence of government trust structure is weaker for online political participation than for offline political participation.

[Contributions] First, building on previous research focused on citizens, this study targets youth as the research subject. Second, this study is the first to explore the impact of government trust structure on political participation and its underlying mechanisms. By linking government trust structure, subjective well-being, and political participation, the study delves into the deeper relationship between government trust structure and political participation. Lastly, existing research has primarily focused on hierarchical government trust and non-institutionalized political participation, lacking a comprehensive perspective on how different government trust structures affect various forms of political participation. On the one hand, measuring hierarchical government trust by the trust difference between central and local governments may overlook the impact of paradoxical government trust on political participation. On the other hand, in the internet era, it is more relevant to classify political participation into online and offline categories rather than the traditional institutionalized and non-institutionalized ones. Therefore, further research is needed to examine how government trust structure influences online and offline political participation.

## Theoretical basis and research hypothesis

### Government trust structure and political participation

Government trust, as a significant element of political support and government legitimacy, is a critical measure of the success or failure of a government [17]. According to the theory of reasoned action, people’s behavior depends on their expectations regarding the consequences of their actions and their evaluation of these consequences. Specifically, a person’s behavioral intentions are determined by the weighted sum of their attitudes toward the behavior and subjective norms. Government trust represents a political attitude, while political participation is a behavior. Public political behavior is influenced by public political attitudes in four key aspects.

Firstly, the theory of reasoned action posits that individuals make rational decisions based on the acquisition of effective information. In the context of political participation, the level of trust citizens have in the government may influence their willingness and ability to access political information. If citizens have high trust in the government, they may be more inclined to actively seek out political information, enabling them to make more informed and rational decisions about their political participation. Secondly, the theory of rational action posits that individuals pursue utility maximization, meaning they choose behaviors that offer the greatest satisfaction or benefit. Utility maximization refers to the tendency of individuals to choose options that are expected to bring the greatest satisfaction or benefit when faced with multiple choices. This decision-making process reflects the consistency of choices, meaning individuals make the most advantageous decisions based on their anticipated utility of various options. According to the theory of utility maximization, individuals evaluate the potential utility of different choices during the decision-making process and select the option that provides the highest level of satisfaction or benefit. This means that utility maximization not only focuses on the absolute utility of choices but also on the consistency of choices, specifically how individuals optimize their decisions to achieve the maximum personal utility. In the context of political participation, citizens may assess the utility of engaging in political activities based on their level of trust in the government. If citizens believe that the government can effectively address social problems, they are more likely to participate in political activities to support societal development and improvement. Thirdly, the theory of rational action emphasizes the behavioral pattern of individuals pursuing profit maximization. In the context of political participation, citizens may assess the impact of government decisions on their own interests based on their trust in the government. If citizens believe that the government will implement policies that align with their interests, they are more likely to actively engage in political activities to protect and promote those interests. Lastly, the theory of reasoned action considers individuals’ attitudes and their approach to handling risks. In political participation, citizens may assess the potential risks and benefits of engaging in political activities based on their level of trust in the government. If citizens believe that the government can manage social affairs in a stable and harmonious manner, they are more likely to participate in political activities to support social stability and development.

Based on variations in public trust levels towards the central government and local governments, the government trust structure can be categorized into four types [10,12], refer to Table 1. The variation in public trust evaluations between the central government and local governments stems from differing perspectives. Some individuals perceive the central and local governments as homogeneous and integral entities, while others see them as distinct hierarchical structures. In general, when disparities exist, it suggests that the public views the central and local governments as distinct hierarchical structures. In this scenario, there are two situations: Situation 1, where the public trusts the central government more than local governments, which is termed as hierarchical government trust, or Type II. Situation 2 involves the public trusting local governments more than the central government, referred to as paradoxical government trust, or Type IV. When there is no difference, it signifies that the public perceives the central and local governments as a homogeneous entity. In such instances, there are also two scenarios: Situation 1, where the public holds equal trust in both the central and local governments, termed as equal trust, or Type I. Situation 2, where the public harbors equal distrust towards the central and local governments, referred to as equal distrust, or Type III.

**Table 1:** Four types of government trust structures.

	Central government trust	
Local government trust	High	Low
High	Equal trust (Type I)	Paradoxical government trust (Type IV)
Low	Hierarchical government trust (Type II)	Equal distrust (Type III)

Political participation is usually conceptualized as public actions aimed at directly or indirectly influencing government decisions, which includes a wide array of activities like voting, campaigning, contacting officials, circulating petitions, displaying stickers, and protesting [18–20]. With the development of the internet, the outreach of political participation is expanding, and the public can participate online, such as contacting elected officials through official websites and social media [21]. Political participation can generally be divided into institutional participation and non-institutional participation. Institutional participation refers to actions taken by citizens within the existing political framework and legal system to express their political demands through formal channels, such as voting, policy consultation and government hearings, joining political parties or civic organizations, and formal petitioning. Non-institutional participation refers to political actions taken outside the formal political system, usually to express protests, exert pressure, or promote social change, such as demonstrations and marches, boycotts, and unlawful protests.

Equal trust holders are considered as “obedient people”, who have the highest level of support for the existing system [13]. Youth with high level trust in the government believe that government will do its best to provide better quality services for citizens, and they also understand government actions and support the government and actively cooperate with the government’s actions. Equal trust holders perceive themselves to be in a highly trustworthy environment and are more likely to engage in institutionalized political participation to express their demands, as they believe the government will respond to their needs. Prior researches find that political trust has a positive effect on institutional political participation, but negative effect on non-institutional political participation [22]. Thus, compared with protest political participation such as marches, demonstrations, and mass events, equal trust holders prefer moderate political participation such as participating in elections and expressing their opinions. The main reason for this is that equal trust holders are more willing to give up their own interests and defend the collective interests when they face the protest political participation. Even if government makes mistakes in the process of implementation, equal trust holders can forgive government and believe that government can accumulate experience from its mistakes to avoid similar mistakes from happening again and improve the administrative efficiency of the government [23]. Equal trust holders are more willing to participate political moderately, giving full play to their own subjective initiative, giving suggestions for government and realizing their own interests. Generally, compared with institutionalized political participation, protest political participation tends to be more costly and risky, and in a relatively stable social environment, equal trust holders basically do not choose adversarial political participation. Therefore, equal trust has a positive effect on political participation.

By the same token, opposite to equal trust holders are equal distrust holders, who have a negative effect on political participation. Equal distrust holders tend to be either politically apathetic or anti-political system, with political apathy being more prevalent. This apathy stems from their deep-seated skepticism toward both central and local governments, leading to a belief that political engagement, whether institutional or non-institutional, yields little to no tangible outcomes. Equal distrust holders often perceive political institutions as ineffective, unresponsive, or even corrupt, reinforcing their reluctance to participate in any form of political action. They consider protest an act of futility, as they believe that no government is likely to take meaningful remedial action in response. In extreme cases, they may fear that engaging in protests or demonstrations carries personal risks, such as imprisonment, injury, or job loss [24–26]. Moreover, the lack of trust in any governing body diminishes their perceived efficacy of both collective and individual political action, further discouraging participation. Additionally, equal distrust holders may experience learned helplessness, a psychological state where individuals, after repeated experiences of political disillusionment or repression, internalize the belief that their actions are powerless to bring about change. This phenomenon not only suppresses political engagement but also fosters a sense of alienation from political discourse and civic life. Consequently, they are less likely to engage even in low-cost political activities, such as voting or signing petitions, as they assume that all political processes are either manipulated or meaningless. Therefore, for equal distrust holders, political participation does not make much sense to address their demands [16]. Instead, they may retreat from political life altogether, leading to a cycle of disengagement that further entrenches their distrust and reinforces systemic stagnation.

If we consider equal trust holders as the control group, paradoxical government trust holders exhibit the most active political participation. These individuals have greater trust in local governments, maintain closer connections with them, interact more frequently, and have a more significant impact on political participation. The reasons for this are multifaceted. Firstly, paradoxical government trust holders apply pressure on both central and local governments through protest participation, but they do not fear that local governments will resort to violence or coercion to suppress them. This confidence stems from their belief that local governments, despite potential inefficiencies, remain responsive and capable of addressing their demands [16]. In contrast, individuals who distrust both central and local governments may perceive protests as futile or even risky, thereby avoiding political engagement altogether. The paradoxical trust structure thus provides a perceived safe space for political participation, where individuals believe that their actions can influence policy outcomes without severe repercussions. Secondly, paradoxical government trust holders are more likely to utilize traditional institutional mechanisms to resolve grievances, such as filing petitions, engaging in mediation or arbitration procedures, or directly contacting government officials to express their opinions. This suggests that their political engagement is not purely adversarial but also operates within established legal and administrative frameworks. Their trust in local governments encourages them to work within the system rather than outside it, distinguishing them from those who engage exclusively in non-institutional participation. Thirdly, paradoxical government trust holders may engage in moderate political participation as a strategic means of interacting with local governments, building political connections, and securing resources. By actively participating in community affairs, attending policy discussions, or joining advisory committees, they enhance their political efficacy, the belief that their actions can influence government decisions. This form of engagement allows them to accumulate social capital, expand their political networks, and gain access to political resources that could benefit their personal or collective interests [27]. Furthermore, their active participation may serve as a mechanism for policy feedback, wherein local governments, recognizing their engagement, adjust governance strategies to maintain public trust and legitimacy. This creates a dynamic interaction between citizens and local authorities, fostering a more participatory political culture. In the long run, such engagement can contribute to incremental institutional reforms, making governance more responsive and inclusive. Therefore, paradoxical government trust holders exhibit a dual engagement strategy, they combine institutional participation with selective non-institutional actions (such as peaceful protests) to amplify their political influence. Unlike equal distrust holders, who may withdraw from political life due to disillusionment, paradoxical trust holders see political engagement as both viable and effective, making them one of the most politically active groups.

Hierarchical government trust holders behave less active than paradoxical government trust holders. Although hierarchical government trust holders do not trust local governments, they trust central government extremely. The trust in central government will give them a sense of merit, that they believe that the dignity of maintaining the central government’s policies deserves protection and praise from the central government. The larger the gap in hierarchical government trust, the more likely individuals are to underestimate the risks of challenging local governments. They may use media to amplify issues, turning personal grievances into public discourse in hopes of drawing the attention of higher authorities to rectify unfavorable situations [14]. Then, protests represent a call by locals to central authorities to seek redress local discontent [28,29], the central government prevents corruption or abuse of power in local governments through an institutionalized inspection system to achieve the public’s expectation [30]. In terms of moderate political participation, individuals who trust the central government may feel that, since local government officials are appointed by the central government and the central government advocates for democracy and is attentive to the people’s needs, participating in politics allows them to both express support for the central government and oversee the local government on its behalf. Consequently, while hierarchical and paradoxical government trusters share similar motivations for resistant political participation, paradoxical government trusters are more motivated than hierarchical trusters when it comes to moderate political participation [16]. As a result, hierarchical government trust holders are less motivated to participate in politics than paradoxical government trust holders.

Accordingly, the study proposes **Hypothesis 1**

Paradoxical government trust holders are the most active in political participation, followed by hierarchical government trust holders, then equal trust holders, finally equal distrust holders.

### Government trust structure and subjective well-being

Subjective well-being refers to the comprehensive evaluation of personal life quality according to the criteria chosen by themselves, including the cognitive dimension of life satisfaction and the emotional dimension of emotional balance [31]. Some scholars refer to life satisfaction and happiness as an equivalent term [32], and consider life satisfaction as the best empirical approximation to the concept of subjective well-being [33]. Living in an environment of high trust can induce a sense of belonging to a community, which is an innate need of human beings, and therefore may strengthen subjective well-being [34]. Evidences illuminate that government trust can improve subjective well-being [35,36]. Equal trust holders have positive expectations regarding the actions of both central and local governments. They believe that these governments will enhance their management and construction capabilities and improve policy implementation efficiency. This, in turn, provides high-quality public services, improves the living environment and quality of life, and reduces uncertainty. As a result, the subjective well-being of equal trust holders is enhanced [37].

Conversely, equal distrust holders do not trust the central or local governments can maintain their life security and represent their interest needs. They tend to be uncertain about the future, which makes them less happy than those who trust others [38]. In an environment of equal distrust towards both central and local governments, young people are likely to develop negative emotions such as complaints and dissatisfaction. This can erode the legitimacy of government administration and heighten the trust crisis. Consequently, subjective well-being is likely to be weakened [39].

If we take equal trust holders as the control group, the paradoxical government trust holders’ subjective well-being is slightly lower in comparison. Since China’s power structure produces different priorities for the central and local government [28]. As the primary implementers of policy guidelines, local governments are closer to young people, interact with them more frequently, and are more directly connected to the quality of their daily lives. Compared to the central government, paradoxical government trust holders have stronger positive expectations regarding the impact of local government actions. They believe that local governments can better represent their interests and address their daily needs, thereby enhancing their personal subjective well-being [40].

Hierarchical government trust holders come second. Compared to local governments, they have stronger positive expectations regarding the impact of central government actions and believe that the central government can significantly improve their lives. However, since the central government primarily sets policy guidelines rather than executing them, its main concern is maintaining regime legitimacy. When tasks are decentralized from the central government to local governments, hierarchical trust holders may not have strong expectations for the effectiveness of local government actions. They may perceive local governments as selectively fulfilling or even neglecting their tasks. Consequently, even if they have strong trust in the central government, their subjective well-being can decrease if policies are poorly implemented or fail to be implemented effectively [41]. Thus, increased trust in local governments tends to enhance youth’s subjective well-being more than trust in the central government.

Accordingly, the study proposes **Hypothesis 2**

Equal trust holders have the strongest subjective well-being, followed by paradoxical government trust, then hierarchical government trust holders, and finally equal distrust holders.

### Subjective well-being and political participation

An infrequent but growing literature propose the opinion that subjective well-being is a cause and political participation is an effect [42]. Regardless of the level of youth subjective well-being, it has a positive effect on political participation. On the one hand, participation can be interpreted as a higher level need, because people will only participate in the political process once their basic survival needs have been met [43]. For example, the public suffering from economic hardship may withdraw from the political process and focus on their survival needs [44]. Thus, as the public’s quality of life improves and their subjective well-being is enhanced, they are likely to pursue higher-level psychological needs, which in turn boosts their enthusiasm, ability, and level of political participation. Previous research has confirmed that increased life satisfaction enhances individual participation in institutional forms of political engagement but does not necessarily contribute to participation in protests [45]. Youth with higher level of subjective well-being indicates that effects of government actions meet or exceed theirs expectations, as well as illustrates that they are more concerned about social and political problems in their communities [46], and may solve these problems and achieve their own interests through political participation [32]. On the other hand, a lower level of subjective well-being indicates that the effects of government actions do not meet adolescents’ expectations, leading to feelings of deprivation and frustration. As these negative feelings accumulate, young people are likely to experience complaints, dissatisfaction, and anger, and may become more inclined to engage in protest-based political participation to challenge the status quo [47], in order to vent their emotions while temporarily regaining a strong mentality and status for their own benefit. Whereas, moderate political participation does not have such a psychological and status compensating effect [48]. In other words, it is life dissatisfaction that strengthens protest activities [32]. Lorenzini further argues that this argument should be limited, as life dissatisfaction affects only the protest potential of specific groups, such as youth, workers, and the middle class, but not the general population. He finds that life dissatisfaction enhances the participation of employed youth in contact-based activities, while life satisfaction encourages unemployed youth to engage in protest activities [29].

Subjective well-being has different effects on political participation across various government trust groups. Individuals with higher subjective well-being tend to have a stronger sense of political efficacy, meaning they believe their political actions can bring about positive change. Therefore, among equal trust holders and hierarchical government trust holders, individuals with higher subjective well-being are more likely to engage in institutional political activities, such as voting, policy consultations, and community participation. Additionally, individuals with higher subjective well-being are more likely to form social connections and are willing to participate in politics to maintain or improve the existing social environment. This trend is particularly evident among hierarchical government trust holders, as their trust in the government leads them to believe that institutional participation is an effective means of achieving political goals.

Among paradoxical government trust holders, individuals with higher subjective well-being may exhibit a dual political participation model: on the one hand, they are willing to express their demands through institutional channels, such as petitions or direct communication with government officials, on the other hand, they may also engage in moderate non-institutional forms of participation, such as social media mobilization or peaceful protests. This is because they trust the responsiveness of local governments but simultaneously seek to apply pressure on the central government to secure more favorable policy support.

For equal distrust holders, the impact of subjective well-being is more complex. Generally, this group has low trust in the government, leading to a lack of political efficacy. Even if they experience high subjective well-being, they may still perceive political participation as ineffective. Furthermore, some equal distrust holders with high subjective well-being may choose political disengagement, meaning they completely withdraw from political activities and instead focus their personal well-being on private life, economic activities, or social networks. In contrast, those with low subjective well-being are more likely to develop political anger, which may drive them toward non-institutional and radical political behaviors, such as protests or boycotts.

Accordingly, the study proposes **Hypothesis 3**:

Subjective well-being positively influences youth political participation.

### Data and variables

#### Date.

The data of this paper comes from the data of the 2018 survey on social awareness of netizens, organized and implemented by Professor Ma D. Y. of Renmin University of China. This survey is conducted by means of an online questionnaire covering questions on political participation, government trust, media use, etc. A total of 5,415 initial samples are obtained. Drawing on Yang and Wang [49], with reference to the age definition of youth (14–35 years old) in the Medium and Long-term Youth Development Plan (2016–2025) issued by the National Bureau of Statistics and the Central Committee and State Council of the Communist Party of China, while confines to the characteristics of the data sample, the sample with the age range of 18–34 years old was retained, and a total of 3217 youth samples are obtained. Considering the purpose of this study, 2814 valid samples are obtained after excluding Hong Kong, Macau, Taiwan or overseas questionnaires and samples with missing values or “don’t know/don’t want to say” in each question.

#### Variable measurement.

The explained variable is the political participation, which is measured jointly by question items of political participation. According to Lin and Yin [50], it is measured by the following question, “How do you usually express your views on political, economic and social issues?” including “posting and replying online”, “Speaking on Weibo and WeChat” and other seven ways to examine political participation (see Table 1). Responses range from 1 (never participation) to 4 (frequent participation), and the seven question items are averaged to measure the level of political participation. The Cronbanch’s Alpha coefficient from the seven items is 0.8572, which indicates a relatively high degree of intrinsic consistency.

Referring to the classification of online political participation and offline political participation [51], which can be seen from Table 2, the average of various political participation of youth range from 2.0561 to 2.6734. The lowest level political participation is “actual actions such as marching and voting”, belonging offline political participation. While the highest level political participation is “chatting with friends offline or holding seminars”, belonging also offline political participation. The mean value of online political participation of 2.6086 is greater than the mean value of offline political participation of 2.3700.

**Table 2:** Descriptive statistics of youth on each political participation question item.

Political participation in each question item	Never participation = 1	Basically not participation = 2	Sometimes participation = 3	Frequent participation = 4	Mean
Posting and replying online	452	844	1113	405	2.5227
Speaking on Weibo and WeChat	352	798	1182	482	2.6375
Participating in discussions in online QQ groups and WeChat groups	339	792	1154	529	2.6656
Chatting with friends offline or holding seminars	322	755	1257	480	2.6734
Writing articles and submitting them to the media	868	977	678	291	2.1393
Communicating privately through emails and chat tools	394	803	1121	496	2.6109
Parching, voting and other practical actions	1091	771	655	297	2.0561

The explaining variable is the government trust structure, including equal trust, equal distrust, paradoxical government trust and hierarchical government trust. The auxiliary explanatory variables are central government trust, local government trust, and central-local government trust gap. According to the actual situation in China, respondents are asked about their degree of central government trust, provincial government trust, township government trust, and village committee trust, in a scale of 1–4 with 1 being “completely distrust” and 4 being “very trust”. As shown in Table 3, youth trust in each political institution is ranked from highest to lowest: central government, provincial government, township government, and village committee, and youth trust in government increases with the rise of government hierarchy, verifying Li’s research [11,12].

**Table 3:** Descriptive statistical analysis of youth trust in all levels of government.

Government trust at all levels	Completely distrust = 1	Not very trust = 2	Compare trust = 3	Very trust = 4	Mean
Central government	103	310	842	1559	3.3706
Provincial Government	113	443	1318	940	3.0963
Township government	269	964	1058	523	2.6521
Village committee	392	996	958	468	2.5338

We obtain another composite variable “local government trust” by taking the average of provincial government trust, township government trust and village committee trust. The Cronbanch’s Alpha coefficient from the three items is 0.7798, which indicates a relatively high degree of intrinsic consistency. Subtracting the value of central government trust from the value of local government trust to another variable “difference in trust between central and local government”, according to the difference value, it is decomposed into four groups of dichotomous variables. The difference value equal to zero, it is divided into equal trust and equal distrust, and the difference value less than zero and greater than zero are divided into paradoxical government trust and hierarchical government trust respectively. The sample sizes of equal trust, equal distrust, paradoxical government trust and hierarchical government trust are 456, 109, 339 and 1910, accounting for 16.19%, 3.87%, 12.05% and 67.87% of the total valid sample sizes respectively. It verifies that hierarchical government trust is the mainstream form of government trust in China [12].

The intermediary variable is subjective well-being. Using for reference of Fu [35], we measure on respondent’s subjective well-being with the question “generally speaking, do you think you are happy now?” There are 5 options, ranging from 1 (very unhappy) to 5 (very happy).

According to Zheng [15], Liu and Peng [52], the control variables include gender, age, urban/rural areas, political affiliation, annual household income, education background, political discussion, political interest, political and social satisfaction, internal efficacy, social trust, official media use, unofficial media use and province.The paper selects “the frequency of watching current affairs news programs on local TV stations” and “the frequency of current affairs analysis reports on CCTV, Xinhua News Agency, and People’s Daily (including microblogs and WeChat public numbers)” items such as measurement. Responses range from 1 (hardly use) to 4 (use almost every day). The average of these two questions are taken as “official media use” variable.The paper selects “the frequency of commercial portals such as Phoenix”, “the frequency of sharing by acquaintances and friends such as WeChat’s circle of friends”, “the frequency of foreign media such as twitter”, “the frequency of reading professional magazines such as South window”, “the frequency of websites such as Tianya community”, “the frequency of self-media such as unofficial WeChat public number” and “the frequency of gossip” items such as measurement. Responses range from 1 (hardly use) to 4 (use almost every day). The average of these seven items are taken as “unofficial media use” variable. Specific descriptions of the variables can be found in Table 4.

**Table 4:** Descriptive statistics of each variable.

Variable	Problem description	Mean	SD	Min	Max
Political participation	The average of question item of political participation	2.4722	0.6889	1	4
Government trust structure	Equal trust	0.1620	0.3686	0	1
	Equal distrust	0.0387	0.1930	0	1
	Paradoxical government trust	0.1205	0.3256	0	1
	Hierarchical government trust	0.6787	0.4670	0	1
Subjective well-being	Generally speaking, do you think you are happy now	4.0679	0.8803	1	5
Gender	Female = 0; Male = 1	0.5227	0.4996	0	1
Age	18-24years old = 1; 25–29years old = 2; 30–34years old = 3	1.7385	0.7470	1	3
Urban and rural areas	Village = 0; City = 1	0.9247	0.2640	0	1
Political affiliation	Non-party member = 0; Party member = 1	0.3326	0.4712	0	1
Annual household income	Less than $50,000 = 1; $50,000-$100,000 = 2; $100,000-$150,000 = 3; $150,000-$200,000 = 4; $200,000-$250,000 = 5; $250,000-$300,000 = 6; $300,000-$350,000 = 7; $350,000-$400,000 = 8; $400,000 or more = 9	3.5750	2.0970	1	9
Education background	Junior high school and below = 1; High school = 2 Specialized, undergraduate = 3; Master’s, doctorate = 4	3.0835	0.6177	1	4
Political discussion	Do you often talk to others about domestic and international political, economic and social issues?	2.7424	0.8318	1	4
Political interest	In general, are you interested in current affairs information?	3.5714	1.1511	1	5
Internal efficacy	I feel I am capable of participating in public affairs	3.7370	0.9850	1	5
Political and social satisfaction	Generally speaking, are you satisfied with the current political and social situation in our country?	4.0988	0.9079	1	5
Social trust	Your attitude towards the majority of people in our society can be trusted	3.6770	1.0166	1	5
Official media use	The average of question item of official media use	2.7877	0.7469	1	4
Unofficial media use	The average of question item of unofficial media use	2.5742	0.6207	1	4
Province	Thirty-one provinces (dummy variables)	9.5789	7.4216	1	31

## Research findings

### Baseline regression results

Table 5 presents the results of the effect of government trust structure on political participation. The results in the first four columns show that equal trust and paradoxical government trust positively affect political participation, with significance at least at the 5% level. Conversely, equal distrust and hierarchical government trust negatively affect political participation, though the effect of equal distrust is not significant. Column (5) uses equal trust as the benchmark and shows that both equal distrust and hierarchical government trust have significant negative effects on political participation, with the coefficient for equal distrust being larger than that for hierarchical government trust. Paradoxical government trust positively affects political participation, but this effect is not significant. This suggests that motivation for political participation decreases from high to low among paradoxical government trust holders, equal trust holders, hierarchical government trust holders, and equal distrust holders. In summary, hypothesis 1 is partially confirmed.

**Table 5:** Baseline regression results of government trust structure and political participation.

	(1)	(2)	(3)	(4)	(5)	(6)	(7)
	Political participation					First re-estimated political participation	Second re-estimated political participation
Equal trust	0.0950^**^						
	(0.0315)						
Equal distrust		-0.0478			-0.1268^**^	-0.1016^*^	-0.2783^**^
		(0.0508)			(0.0576)	(0.0590)	(0.0857)
Paradoxical government trust			0.1282^***^		0.0311	0.0354	0.0057
			(0.0300)		(0.0400)	(0.0400)	(0.0686)
Hierarchical government trust				-0.1174^***^	-0.1213^***^	-0.0792^***^	-0.3735^***^
				(0.0217)	(0.0316)	(0.0314)	(0.0511)
Gender	0.0251	0.0244	0.0243	0.0254	0.0250	0.0215	0.0463
	(0.0194)	(0.0197)	(0.0191)	(0.0191)	(0.0194)	(0.0192)	(0.0341)
Age	-0.0085	-0.0099	-0.0077	-0.0062	-0.0055	-0.0006	-0.0353
	(0.0134)	(0.0127)	(0.0131)	(0.0131)	(0.0132)	(0.0136)	(0.0215)
Urban and rural areas	0.0092	0.0024	0.0012	0.0101	0.0091	0.0373	-0.1600^**^
	(0.0397)	(0.0405)	(0.0380)	(0.0380)	(0.0402)	(0.0389)	(0.0687)
Political affiliation	0.0088	0.0111	0.0088	0.0056	0.0061	0.0091	-0.0121
	(0.0206)	(0.0215)	(0.0211)	(0.0211)	(0.0213)	(0.0206)	(0.0360)
Annual household income	-0.0113^**^	-.01330^**^	-0.0125^**^	-0.0100^*^	-0.0099^*^	-0.0062	-0.0318^***^
	(0.0052)	(0.0053)	(0.0051)	(0.0051)	(0.0052)	(0.0054)	(0.0086)
Education background	-0.0357^**^	-0.0304^*^	-0.0293^*^	-0.0367^**^	-0.0349^*^	-0.0301^*^	-0.0636^**^
	(0.0180)	(0.0178)	(0.0168)	(0.0168)	(0.0178)	(0.0179)	(0.0291)
Political discussion	0.1528^***^	0.1514^***^	0.1548^***^	0.1564^***^	0.1569^***^	0.1734^***^	0.0583^**^
	(0.0155)	(0.0150)	(0.0131)	(0.0131)	(0.0158)	(0.0161)	(0.0239)
Political interest	-0.0347^**^	-0.0353^**^	-0.0315^**^	-0.0311^**^	-0.0301^**^	-0.0253^**^	-0.0587^***^
	(0.0109)	(0.0112)	(0.0097)	(0.0097)	(0.0108)	(0.0112)	(0.0166)
Internal efficacy	0.0405^***^	0.0441^***^	0.0456^***^	0.0407^***^	0.0414^***^	0.0442^***^	0.0244
	(0.0107)	(0.0108)	(0.0103)	(0.0103)	(0.0107)	(0.0109)	(0.0186)
Political And social satisfaction	0.0678^***^	0.0723^***^	0.0774^***^	0.0743^***^	0.0692^***^	0.0579^***^	0.1375^***^
	(0.0124)	(0.0117)	(0.0123)	(0.0122)	(0.0126)	(0.0129)	(0.0210)
Social trust	0.0081	0.0111	0.0139	0.0103	0.0104	0.0139	-0.0106
	(0.0105)	(0.0106)	(0.0102)	(0.0102)	(0.0108)	(0.0106)	(0.0177)
Official media use	0.0059	0.0010	0.0046	0.0127	0.0089	0.0245	-0.0849^**^
	(0.0175)	(0.0178)	(0.0170)	(0.0171)	(0.0178)	(0.0180)	(0.0277)
Unofficial media use	0.6099^***^	0.6209^***^	0.6115^***^	0.5968^***^	0.5982^***^	0.5818^***^	0.6962^***^
	(0.0213)	(0.0210)	(0.0204)	(0.0207)	(0.0217)	(0.0223)	(0.0345)
Province	No	Yes	Yes	Yes	Yes	Yes	Yes
R^2^	0.4749	0.4729	0.4762	0.4782	0.4796	0.4698	0.3166
N	2814	2814	2814	2814	2814	2814	2814

Note. (1) The standardized coefficients in cells, standard errors in parentheses. (2) *p < 0.10, **p < 0.05, ***p < 0.01.

The baseline regression is more similar to the findings obtained from existing studies on government trust structures on electoral political participation [16]. Among the political participation questions, only one question is related to protest political participation, and it is combined with electoral political participation as one question, the rest of the political participation questions are all moderate political participation. Therefore, this question item is excluded and the mean of the remaining six political participation questions is calculated as the first reestimated political participation. The result is shown in column (6) and is consistent with the result in column (5). Furthermore, moderate political participation is consistently more likely to occur than protest political participation. Among the political participation items, only those encompassing both electoral and protest political participation are retained for the second re-estimation of political participation. The results, shown in column (7), remain largely consistent with those in column (5). Notably, the estimated coefficients for hierarchical government trust exceed those for equal distrust. This suggests that, as the scope of political participation is narrowed, the effect of protest political participation, previously overshadowed by moderate political participation, becomes more pronounced.

### Robustness tests

This paper tests the robustness of the benchmark regression results through the effect of government trust on political participation. Results in the first three columns of Table 6 show that the sum of trust between central and local government strengthens political participation, the difference of trust between central and local government weakens political participation. Results in the last five columns show that central government trust and the difference of trust between central and local government weaken political participation, while local government trust, and the interaction term of local government trust and central government trust strengthen political participation. The above results reveal that political participation will increase with the increase of trust in government, especially the increase of trust in local government and the decrease of trust gap between central and local government. It can be roughly deduced that, equal trust and paradoxical government trust strengthen political participation, equal distrust and hierarchical government trust weaken political participation. In summary, the robustness of government trust structure to political participation passes the test.

**Table 6:** Robustness test.

	(1)	(2)	(3)	(4)	(5)	(6)	(7)	(8)
The sum of trust between central and local government	0.0420^**^		0.0209					
	(0.0188)		(0.0183)					
The difference of trust between central and local government		-0.0829^***^	-0.0801^***^					-0.0837^***^
		(0.0120)	(0.0119)					(0.0124)
Central government trust				-0.0470^***^		-0.0749^***^	-0.1534^**^	
				(0.0129)		(0.0136)	(0.0448)	
Local government trust					0.0661^***^	0.0958^***^	-0.0160	
					(0.0168)	(0.0175)	(0.0619)	
Central government trust ×Local government trust							0.0323^*^	0.0045
							(0.0177)	(0.0031)
Control variables	Yes	Yes	Yes	Yes	Yes	Yes	Yes	Yes
R^2^	0.4738	0.4810	0.4813	0.4750	0.4761	0.4813	0.4820	0.4814
N	2814	2814	2814	2814	2814	2814	2814	2814

Note. (1) The standardized coefficients in cells, standard errors in parentheses. (2) *p < 0.10, **p < 0.05, ***p < 0.01.

### Mediated test of subjective well-being

Table 7 reports the results of the test for mediating effects of subjective well-being. Results in the first four columns show that equal trust significantly and positively affects subjective well-being, hierarchical government trust significantly and negatively affects subjective well-being, and the remaining two government trust structures do not have significant effects on subjective well-being. In column (5), with equal trust as the benchmark, paradoxical government trust, hierarchical government trust, and equal distrust negatively affect subjective well-being from low to high, and only the coefficient of hierarchical government trust is significant, hypothesis 2 is partially confirmed. The result in column (6) shows that the effect of government trust structure on political participation is consistent with the result of the benchmark regression, and subjective well-being positively affects political participation, hypothesis 3 is confirmed. Subjective well-being mediates the effect of equal trust and hierarchical government trust on political participation.

**Table 7:** Mediation tests for subjective well-being.

	(1)	(2)	(3)	(4)	(5)	(6)	(7)	(8)
	Subjective well-being					Political participation	Subjective well-being	
Equal trust	0.0659^**^							
	(0.0301)							
Equal distrust		-0.0484			-0.1037	-0.1227^**^		
		(0.0743)			(0.0784)	(0.0573)		
Paradoxical government trust			0.0574		-0.0060	0.0314		
			(0.0425)		(0.0471)	(0.0400)		
Hierarchical government trust				-0.0621^**^	-0.0774^**^	-0.1182^***^		
				(0.0268)	(0.0299)	(0.0311)		
Subjective well-being						0.0397^**^		
						(0.0143)		
Central government trust							0.0002	
							(0.0199)	
Local government trust							0.1010^***^	
							(0.0218)	
The sum of trust between central and local government								0.1012^***^
								(0.0240)
The difference of trust between central and local government								-0.0251
								(0.0168)
Control variables	Yes	Yes	Yes	Yes	Yes	Yes	Yes	Yes
R^2^	0.5247	0.5242	0.5245	0.5250	0.5254	0.4726	0.5289	0.5289
N	2814	2814	2814	2814	2814	2814	2814	2814

Note. (1) The standardized coefficients in cells, standard errors in parentheses. (2) *p < 0.10, **p < 0.05, ***p < 0.01.

Equal distrust and paradoxical government trust are not significant for subjective well-being. We provide two explanations, first, equal distrust holders believe that their subjective well-being have nothing to do with whether they trust the central and local government or not, but comes more from their own efforts to improve their own living standards and enhance their subjective well-being through employment and remuneration for their labor. Second, paradoxical government trust holders trust local government more and believe that local government can satisfy their own interests, and their subjective well-being is weakened compared to equal trust holders, but the weakening is smaller, so there is no significant difference between them and equal trust holders. To preliminarily test the conjecture of the second point, the results in columns (7) and (8) show that the difference of trust between central and local government is insignificant for subjective well-being, while local government trust and the sum of trust between central and local government are significantly positive for subjective well-being. This suggests that subjective well-being will increase as trust in government increases, especially as trust in local government increases and as the trust gap between central and local government decreases.

### The effect of government trust structure on different types of political participation

Table 8 reports the impact of government trust structure on online political participation and offline political participation. The results indicate that, using equal trust as the benchmark, the negative impact of equal distrust and hierarchical government trust on online political participation is smaller than their negative impact on offline political participation, whereas the positive impact of paradoxical government trust on online political participation is greater than its positive impact on offline political participation. In online political participation, using equal trust as the benchmark, the coefficients of equal distrust, paradoxical government trust, and hierarchical government trust are all insignificant. Additionally, in offline political participation, the negative impact of hierarchical government trust is stronger than that of equal distrust, while the coefficient of paradoxical government trust remains insignificant.

**Table 8:** Mechanisms of government trust structure on online political participation and offline political participation.

	(1)	(2)	(3)	(4)	(5)	(6)	(7)	(8)	(9)	(10)
	Online political participation					Offline political participation				
Equal trust	0.0217					0.1501^***^				
	(0.0347)					(0.0326)				
Equal distrust		-0.0625			-0.0779		-0.0368			-0.1635^**^
		(0.0664)			(0.0707)		(0.0530)			(0.0588)
Paradoxical government trust			0.0729^**^		0.0433			0.1696^***^		0.0220
			(0.0351)		(0.0462)			(0.0325)		(0.0426)
Hierarchical government trust				-0.0404	-0.0335				-0.1751^***^	-0.1871^***^
				(0.0262)	(0.0352)				(0.0240)	(0.0333)
Control variables	Yes	Yes	Yes	Yes	Yes	Yes	Yes	Yes	Yes	Yes
R^2^	0.3786	0.3787	0.3794	0.3790	0.3797	0.4455	0.4407	0.4459	0.4514	0.4532
N	2814	2814	2814	2814	2814	2814	2814	2814	2814	2814

Note. (1) The standardized coefficients in cells, standard errors in parentheses. (2) *p < 0.10, **p < 0.05, ***p < 0.01.

## Conclusion and discussion

Political participation plays an important and positive role in China’s political and social development [53]. This paper empirically explores the impact of government trust structure of youth netizens on political participation and its mechanism of action with the help of data from the 2018 Social Awareness Survey of netizens, and draws the following basic conclusions. Firstly, paradoxical government trust holders are the most active political participants, followed by equal trust holders, then hierarchical government trust holders, and finally equal distrust holders. Secondly, the subjective well-being of equal trust holders, paradoxical government trust holders, hierarchical government trust holders, and equal distrust holders decrease from high to low. Thirdly, government trust structure has heterogeneous effects on online and offline political participation. Specifically, the negative impact of equal distrust and hierarchical government trust on online political participation is smaller than their negative impact on offline political participation, whereas the positive impact of paradoxical government trust on online political participation is greater than its positive impact on offline political participation, the effect of government trust structure on online political participation is weaker than its effect on offline political participation.

In response to the above findings, the following two points deserve further discussion. Firstly, Central government trust significantly exerts negative effect on political participation. The sample data reveals that the average of trust in the central government for youth is higher at 3.3706 than the average of political participation at 2.4722, which reflects the coexistence of high level of central government trust and low level of political participation. Youth’s trust in the central government is more emotional in origin. To some extent, it can be interpreted as a regime based trust, which has a debilitating effect on protest political participation [54], thereby inhibiting political participation. In addition, youth with a high level of trust in the central government will believe that even if they do not engage in political participation, the central government will urge the local government to protect their rights and interests.

Secondly, the government trust structure has heterogeneous effects on online and offline political participation. Equal distrust and hierarchical government trust have a weaker influence on online political participation compared to offline political participation, whereas paradoxical government trust exhibits the opposite pattern, with individuals more inclined to engage in online rather than offline political activities. This distinction can be attributed to differences in political efficacy, risk perception, and the perceived effectiveness of different forms of participation. First, individuals with equal distrust and hierarchical government trust tend to be less enthusiastic about political participation in general, whether online or offline. However, when they do participate, they find offline political activities more effective in influencing policy outcomes, as they perceive online participation to be largely symbolic and less impactful. Equal distrust holders, due to their deep skepticism toward both central and local governments, often lack political efficacy, leading to an overall disengagement from political affairs. Hierarchical government trust holders, on the other hand, believe in the legitimacy and responsiveness of governmental institutions, making them more inclined toward formal offline participation, such as voting, policy consultations, and community engagement, rather than online activism. Second, hierarchical government trust holders, despite being active in both online and offline political participation, exhibit a stronger preference for contentious offline engagement, such as demonstrations, petitions, and direct interactions with policymakers. This supports the notion that hierarchical trust serves as a mobilizing force, encouraging individuals to engage in political expression through structured, yet sometimes confrontational, offline channels where they believe their voices will be heard and considered. Third, paradoxical government trust holders, who trust local governments while distrusting the central government, strategically favor online political participation over offline protests. While they believe that local governments are responsive and unlikely to use force against them, they recognize that large-scale offline protests could pose a risk to the local governments they trust. As a result, they prefer online platforms as a middle-ground strategy, allowing them to express political demands without directly challenging local authorities. The empirical results further support these arguments. In online political participation, the coefficients for equal distrust, paradoxical government trust, and hierarchical government trust are all insignificant, suggesting that online engagement is relatively uniform across trust structures. However, the coefficient for equal distrust is slightly larger than that for hierarchical government trust, indicating that even among disengaged groups, equal distrust holders exhibit slightly higher online activity, possibly as a way to vent frustrations in a low-cost and low-risk environment. In contrast, offline political participation shows a different trend, with hierarchical government trust exerting a stronger influence, reinforcing the idea that trust in institutions encourages direct political engagement. The nature of online participation, characterized by low cost, convenience, and widespread adoption among younger demographics, contributes to less variation in engagement levels across different trust structures. Equal distrust holders remain largely disengaged in both online and offline political participation. Although they exhibit slightly higher enthusiasm for offline protests compared to hierarchical trust holders, their overall engagement remains limited, reflecting their deep political disillusionment and skepticism about the effectiveness of participation. These findings highlight the complex interplay between government trust, participation modes, and individual strategic considerations, demonstrating that while trust fosters engagement in institutionalized political activities, it also shapes whether individuals choose to express their demands in the online or offline sphere.

Based on the research findings, this paper proposes the following policy recommendations: the government should adopt different strategies for youth with different government trust structures. For equal trust holders, the government should optimize institutional participation channels and encourage active civic engagement by providing platforms such as youth policy consultations, government-youth dialogues, and social organization programs. Offering incentives like social practice opportunities and public governance awards can further stimulate their interest in public affairs. Strengthening political socialization education will enhance their understanding of policymaking and governance, fostering deeper participation. For equal distrust holders, the priority should be rebuilding trust, lowering participation barriers, and enhancing political efficacy. This requires increasing government transparency, strengthening anti-corruption efforts, and improving responsiveness to public concerns. Expanding diverse participation channels, such as social media discussions, youth councils, and community governance. Additionally, promoting political education and critical thinking can help restore confidence and encourage participation. For hierarchical government trust holders, the government should strengthen institutional channels and government-youth interactions through policy consultations and public governance collaborations. Encouraging their involvement in local governance, such as community deliberations and grassroots self-governance, can foster trust in institutions while reducing over-reliance on local governments. For paradoxical government trust holders, the focus should be on enhancing governance transparency, improving policy communication, and promoting institutionalized participation. Strengthening local government transparency and public interaction mechanisms can encourage constructive engagement. Guiding them toward formal channels like petitions, negotiations, and citizen proposals will help them see government responsiveness, reducing skepticism toward the central government and increasing overall political efficacy.

This paper constructs an analytical framework between government trust structure, subjective well-being, and political participation through logical deduction with youth as the research target. It further analyzes the relationship between government trust structure and online political participation and offline political participation. There are some expansions in the research object and research content, but shortages still exist. Firstly, this study focuses on the youth population, and the sample excludes data from Hong Kong, Macau, and Taiwan. Therefore, whether the research findings are applicable to older populations and groups in Hong Kong, Macau, and Taiwan requires further investigation. Secondly, even though many control variables are added, they are limited to the specificity of the respective variables and fail to find a suitable instrumental variable to solve the endogeneity problem. Thirdly, the study on the relationship between government trust structure and political participation among different government levels needs further exploration with the subsequent addition of city/county government trust.
